# Loss of galactan synthesis in pea (*Pisum sativum*) causes defects in organ expansion and is associated with increased extensin content

**DOI:** 10.1007/s11103-026-01759-x

**Published:** 2026-09-09

**Authors:** Xiaoyuan Guo, Ajay Kumar, Ida Elisabeth Johansen, Jesper Harholt, William G. T. Willats, Peter Ulvskov, Jozef Mravec

**Affiliations:** 1 Department of Plant and Environmental Sciences, University of Copenhagen, Thorvaldsensvej, 40, 1871 Frederiksberg C, Denmark; 2 Institute of Plant Genetics and Biotechnology, Plant Science and Biodiversity Centre, Slovak Academy of Sciences, Akademická 2, 949 01 Nitra, Slovakia; 3 Carlsberg Research Laboratory, J.C. Jacobsens Gade 4, 1799 Copenhagen V, Denmark; 4 Present Address: Novonesis, Biologiens Vej 2, 2800 Kongens Lyngby, Denmark; 5 School of Agriculture, Food and Rural Development, Newcastle University, Newcastle Upon Tyne, UK

**Keywords:** *Pisum sativum*, Cell elongation, Galactan, Extensin, Virus-induced gene silencing, Microarray polymer profiling, Monoclonal antibody, Gene expression

## Abstract

Pectic galactan is a rhamnogalacturonan-I side chain, composed of β-(1→4)-linked D-galactose residues. Its multifunctional role in cell wall assembly and properties has been proposed in various developmental contexts. In this study, we investigated the role of galactan in pea (*Pisum sativum syn. Lathyrus oleraceus*). First, we used glycan profiling to examine stems with different auxin-induced elongation rates for changes in cell wall composition. The galactan epitopes, recognised with the LM5 monoclonal antibody, were more abundant in the convex, more elongated part of the pea stem. This finding was supported by monosaccharide composition analysis and epitope detection chromatography (EDC). To evaluate the impact of disrupting galactan synthesis on pea development, we used Virus-Induced Gene Silencing (VIGS) to downregulate the auxin-inducible pea ortholog of Arabidopsis β-1,4-galactosyltransferases of the GT92 family (*PsGALS3*). Plants with silenced *PsGALS3* exhibited severe defects in elongation and vegetative organ growth. Microarray polymer profiling (MAPP) revealed that, alongside the reduction in galactan epitopes, the cell wall proteoglycan extensin content increased in these plants, as confirmed by in situ immunohistological analysis. Our results provide new evidence linking galactan to cell and organ expansion.

**Supplementary Information:**

The online version contains supplementary material available at https://doi.org/10.1007/s11103-026-01759-x.

## Key message

Auxin-driven tropic-like responses and virus-induced silencing of the auxin-inducible pea orthologue of the galactan synthase gene *PsGALS3* indicate that cell wall galactan is required for organ expansion.

**Supplementary Information:**

The online version contains supplementary material available at https://doi.org/10.1007/s11103-026-01759-x.

## Introduction

The plant cell wall is a distinctive feature that encases and supports the cell, serving as the main barrier against pathogens and environmental stressors (Geitman, 2023; Cosgrove 2024). Its diverse composition and complex 3D structure enable the wall to participate in various cellular functions (Zhang et al. 2025). Cell elongation is essential for plant development and growth. For cells to expand, the relatively rigid extraprotoplasmic structure must be adjusted; the degree and type of these adjustments vary depending on the cellular and developmental context (Labavitch 1981; Cosgrove 2024).

The plant hormone auxin, primarily in the form of indole−3-acetic acid (IAA), plays a crucial role in developmental and directional elongation during tropic responses and generating asymmetries (Du et al. 2020; Jonsson et al. 2025). It has long been understood that auxin activity is mediated by modulation of apoplastic pH and the dynamic interactions of glucan polymers, as described by the acid growth hypothesis (Hager et al. 1971; Labavitch and Ray 1974; Kumar et al. 2025). Besides pH changes, cell wall components undergo compositional and structural dynamics; for example, the complexity of xyloglucan is regulated by auxin during different elongation programs (Velasquez et al. 2021; Aryal et al. 2020). The roles of other cell wall components in elongation have been suggested but remain poorly understood. This is especially true for pectic glycans and cell wall glycoproteins. Galactan, predominantly composed of linear β-(1→4)-linked D-galactose residues, is one of the major side chains of rhamnogalacturonan-I (RG-I), together with (1→5)-α-L-arabinan and arabinogalactans (AGs) classified into type I (AG-I), which consists of a β-(1→4)-galactan backbone substituted with arabinose residues, and the more complex type II (AG-II), characterised by a β-(1→3)-galactan backbone with β-(1→6)-galactan side chains further decorated with additional monosaccharides. In addition, RG-I may contain minor side chains and substitutions further contributing to its structural diversity (Kaczmarska et al. 2022; Anderson and Pelloux 2025).

RG-I is connected to homogalacturonan (HG) at least at the reducing end of the backbone but probably at both ends (Coenen et al. 2007). However, RG-I is also involved in supramolecular complexes in which covalent linkages between RG-I, AGP, and xylan have been demonstrated (Tan et al. 2013). Galactan side chains play an important architectural role in secondary walls, where galactans appear to compete with xyloglucan for binding to cellulose microfibrils (Gao et al. 2023). RG-I side chains are modulated in primary walls. These components are upregulated during the transition from cell proliferation to cell elongation (Catalá et al. 1997; Willats et al. 1999), as observed in suspension-cultured carrot cells. Furthermore, previous in situ localisation studies have shown that galactan is restricted to the transition zone of the Arabidopsis root tip during rapid cell elongation (McCartney et al. 2003; Wilson et al. 2015).

Rapid turnover of the pool of galactose in the wall is characteristic of the elongation zone, but the rate of turnover was not found to change upon auxin stimulation of cell expansion (Labavitch and Ray 1974). The elongation zone is characterised by a higher level of cell wall hydration than in cells in which expansion has ceased (Goldberg et al. 1989); the length of galactan side chains has also been implicated in both hydration and the mechanical properties of cell walls (Ulvskov et al. 2005). The lobing observed in many epidermal anticlinal cell walls appears to depend on local differences in mechanical properties, mediated by differential galactan deposition (Majda et al. 2017).

Galactan is synthesised in the Golgi apparatus by β-1,4-galactosyltransferases (GALS) of the GT92 family (Amos and Mohnen 2019). These glycosyltransferases have been identified in Arabidopsis and have been characterised both enzymatically and structurally (Liwanag et al. 2012; Ebert et al. 2018; Laursen et al. 2018; Prabhakar et al. 2023). Interestingly, Arabidopsis *gals* single and multiple mutants do not exhibit any striking developmental phenotypes (Liwanag et al. 2012; Ebert et al. 2018; Prabhakar et al. 2023) besides the altered response to salt stress (Yan et al. 2021) and exhibit reduced acquisition of freezing tolerance during cold acclimation (Takahashi et al. 2024).

Galactan side-chain initiation and elongation are unlikely to be catalysed solely by GALS, as early biochemical studies suggested the involvement of multiple galactosyltransferase activities (Goubet and Morvan 1994; Geshi et al. 2002). Consistent with this, a rhamnogalacturonan I galactosyltransferase activity capable of transferring galactose to rhamnose residues of RG-I backbone oligosaccharides has been identified in mung bean (Matsumoto et al. 2019). Thus, although GALS is responsible for galactan elongation, the number and identity of the additional activities required for galactan side-chain initiation and assembly remain unknown.

In our study, we used pea (*Pisum sativum* or recently introduced syn. *Lathyrus oleraceus*), a classic model for auxin-mediated control of cell elongation (Matchett and Nance 1962; Labavitch and Ray 1974; Gilkes and Hall 1977; Bret-Harte and Talbott 1993; Sauer et al. 2006). One advantage of pea is that it is highly amenable to experimental manipulation with auxin and provides ample material for analysis of cell wall composition. Additionally, a well-established protocol for virus-induced gene silencing (VIGS) is available (Grønlund et al. 2010). This provided an opportunity to investigate the roles of different cell wall components in elongation. Our results demonstrate that galactan accumulation is enhanced in cells undergoing auxin-mediated elongation and that *PsGALS3* expression is induced by externally applied auxin. In contrast to *Arabidopsis*, disruption of galactan synthesis in pea leads to distinct developmental phenotypes and is accompanied by altered abundance of other cell wall components, particularly extensin-related epitopes.

## Results

### Galactan epitopes are regulated in IAA-induced differential elongation of the stem

**Fig. 1:**
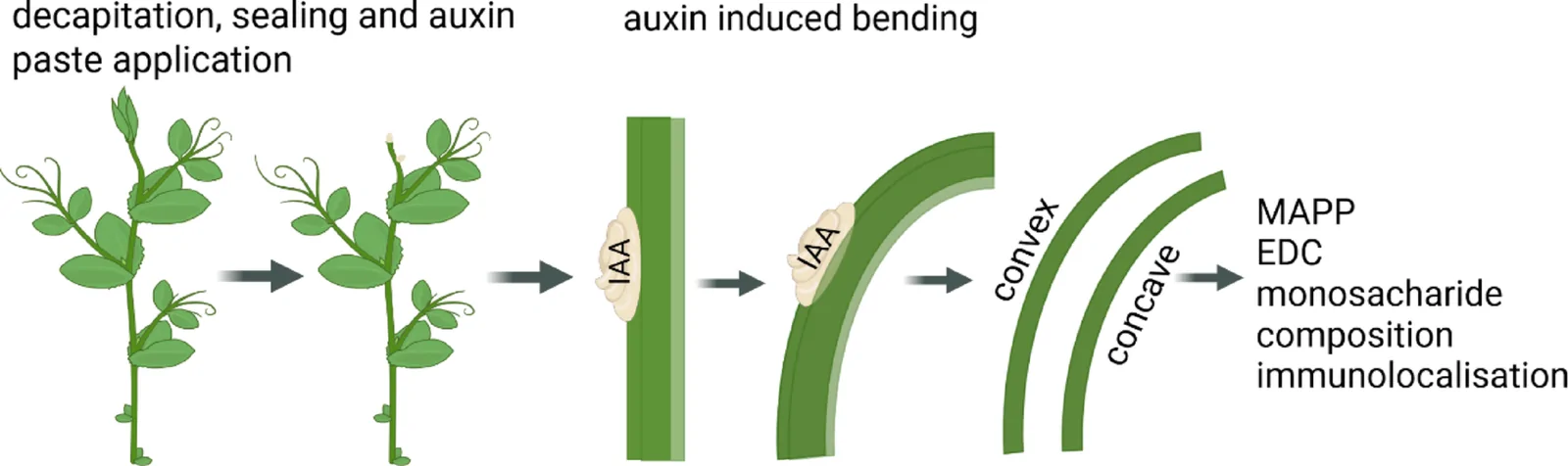
Diagram of auxin-induced bending in pea stems. The pea plants were decapitated, and the wound was sealed with pure lanolin. The lanolin paste containing IAA was applied laterally. The convex and concave sites were dissected and analysed using various methods

We previously reported an experimental setup that induces pea internode curvature, mimicking tropic responses, by the unilateral application of an auxin-containing lanolin paste. This setup enables compositional analysis of cell wall dynamics underlying auxin-mediated differential organ elongation (Velasquez et al. 2021). Unlike the traditional approach of incubating excised pea stem segments directly in auxin-supplemented solutions, this arrangement enables monitoring changes in a more native context. Applying lanolin paste containing IAA to one side of the decapitated pea stem induces differential elongation and curvature after three days (Fig. 1). Applying pure lanolin paste served as a control. The convex (more elongated) and concave (less elongated) parts are dissected with a razor blade and analysed using biochemical and immunological approaches.

**Fig. 2:**
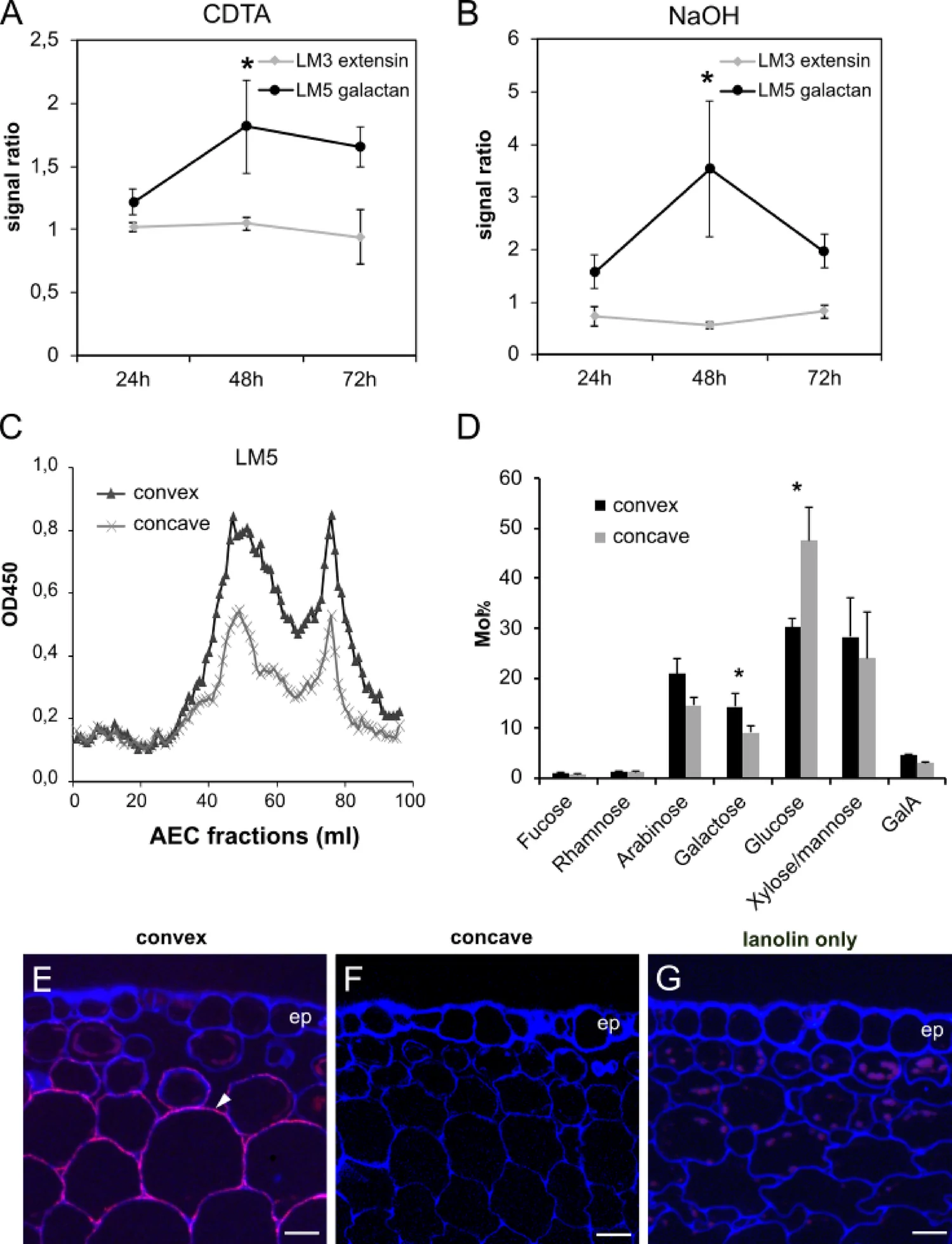
Changes in galactan content in auxin-induced curvature in pea stems. (**A**, **B**) Relative comparison between the concave (shorter) and convex (longer) parts of auxin-induced curvature. LM5 and LM3 binding on extracts from the opposing sides, showing relative amounts of cell wall epitopes detected by specific monoclonal antibodies on different parts of curvature. **A** CDTA extraction is rich in pectic and easily extractable epitopes, while **B** NaOH extraction contains abundant hemicellulose and tightly bound polymers. Values are means of eight biological replicates. **C** Analysis of the CDTA extracts with EDC. The chromatogram for LM5 epitope detection indicates similar structural features of the galactan on opposite sides. The experiment was performed with three biological replicates, with comparable results. **D** Monosaccharide composition analysis of AIR derived from curvature confirms an increase in galactose on the convex side compared to the concave side (mean ± SD of three biological replicates; *P < 0.05, t-test). **E–G** Immunolocalisation of LM5 epitopes in the resin sections through convex (E) and concave (F) sites of the IAA-stimulated internode curvature and lanolin-only control (G). Upregulation of the LM5 epitopes (red, arrowhead) is visible in the cell wall of cortical tissue in the convex site. Note the more expanded cortical cells on the convex side. The cell wall is counterstained with Calcofluor White (blue signal). Scale bars = 10 μm

We previously reported changes in xyloglucan epitopes (Velasquez et al. 2021). Using the same setup, we now analysed the gradual changes in the cell wall composition of opposing curvature regions at three time points—24, 48, and 72 h—using monoclonal Microarray Polymer Profiling (MAPP; Bakshani et al. 2023), previously also known as Comprehensive Microarray Polymer Profiling (CoMPP; Moller et al. 2007). This involved (1) CDTA-chelate-assisted extraction to liberate more soluble cell wall components, and (2) NaOH extraction, which solubilises more recalcitrant cell wall, especially hemicellulosic components. Both extracts were printed onto nitrocellulose arrays, which were then probed with a panel of 13 anti-glycan monoclonal antibodies (listed in Supplementary Table 2). The resulting quantification showed that among the 13 epitopes, galactan epitopes (recognised by the LM5 mAb; Jones et al. 1997) were upregulated in the more elongated, convex part (Fig. 2A, B). The signal ratio between convex and concave sites increased gradually, while extensin epitope levels (recognised by LM3 mAbs) remained relatively stable. The signal intensity of LM5 in the stems treated with pure lanolin paste was similar to that at the concave site (± 3%), confirming that the changes were IAA-triggered. The immuno-glycan profiles indicate that auxin application on the stem induces compositional changes in the affected cell walls, including galactan, which is upregulated in the elongation regions and reaches a maximum at 48 h after IAA application.

### Epitope detection chromatography and monosaccharide composition analysis correlate with MAPP profiling data

To determine whether the variations in LM5 epitope abundance detected with MAPP are due to quantitative differences or structural changes in galactan chains or their linkages to other cell wall components, we used epitope detection chromatography (EDC), which combines anion-exchange chromatography with ELISA (Cornuault et al. 2014). The EDC profiles of LM5 epitopes from convex and concave regions were similar, differing mainly in relative abundance (Fig. 2C). This suggests that the differences are likely quantitative rather than reflecting modifications in the galactan structures that contain epitopes recognised by the LM5 mAb. Consistent with this, monosaccharide composition analysis of AIR using HPLC revealed increased levels of galactose in the convex part of the pea stem compared to the control (Fig. 2D). Conversely, a marked reduction in glucose abundance was observed. Because crystalline cellulose is not hydrolysed by TFA under the conditions used, this decrease is most likely attributable to glucose derived from xyloglucan rather than cellulose. The in vitro observations were confirmed by an in situ detection of the galactan epitopes on cross sections of auxin-stimulated and non-stimulated stems (Fig. 2E–G). The LM5 epitope was found in the cell walls of auxin-engorged cells in the cortical tissue’s convex site but was nearly absent in the concave part.

### Identification of putative pea β-1, 4-galactosyltransferases

**Fig. 3:**
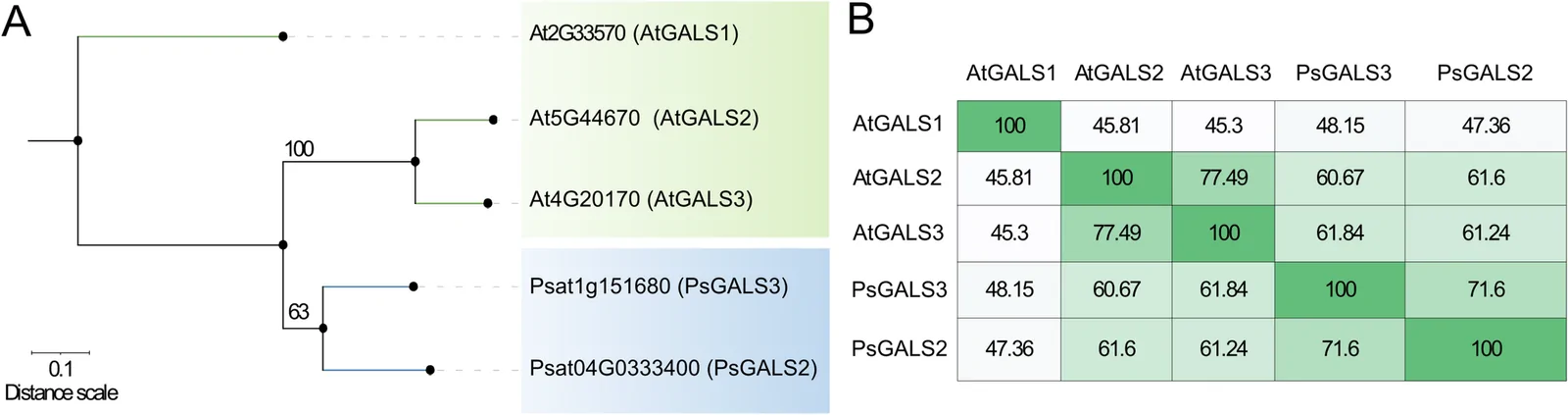
Phylogenetic tree and heat map of sequence identity for GT92 GALS proteins. **A** The Maximum likelihood tree of Arabidopsis AtGALS1, AtGALS2, AtGALS3, and pea homologs PsGALS3 and PsGALS2. The analysis uses the (Jones-Taylor-Thornton) JTT substitution model, and bootstrap values of 50% or higher are shown. The tree is scaled, with branch lengths indicating substitutions per site. **B** Pairwise amino acid sequence identities (%) were calculated for AtGALS1, AtGALS2, AtGALS3, and pea homologs PsGALS3 and PsGALS2. Darker green hues show higher identity scores, lighter shades represent lower scores, and white indicates the lowest score. The diagonal values (100%) represent self-identity

To identify targets for functional analysis, we searched public databases for orthologs of Arabidopsis β-1,4-galactan synthases (Liwanag et al. 2012; Ebert et al. 2018; Yan et al. 2021). Only two were identified: Psat1g151680 and Psat04G0333400. Both exhibit approximately 61% amino acid sequence identity with At5G44670 (AtGALS2) and At4G20170 (AtGALS3). Compared to each other, pea GALS share 71.6% sequence identity (Fig. 3). Psat1g151680 (hereafter designated as *PsGALS3*) is located on linkage group 1LG6 within the *Pisum sativum* Cameor v1a reference genome, specifically at positions 299,495,892 to 299,499,832 bp on the forward strand. This gene is 2,465 bp and encodes a 515-amino-acid-long protein. In comparison, Psat04G0333400 (hereafter designated as *PsGALS2*) is located on chromosome 4 of *Pisum sativum* var. ZW6 v1.0, spanning positions 248,077,528 to 248,080,555 bp on the reverse strand. It spans 3027 bp and encodes a 495-amino-acid protein.

In the phylogenetic analysis of GT92 GALS proteins, Arabidopsis and pea clearly cluster into separate groups (Fig. 3A). In Arabidopsis, AtGALS2 and AtGALS3 form a strongly supported subclade, indicating these two proteins are very similar. In contrast, AtGALS1 branches off separately and is more divergent from AtGALS2 and AtGALS3, suggesting functional or evolutionary differences. The two pea paralogs form a distinct, legume-specific group. While PsGALS3 and PsGALS2 have moderate bootstrap support compared to AtGALS, this suggests possible species-specific duplication or diversification within pea. The clear separation between Arabidopsis and pea clusters matches the expected evolutionary distances between Brassicaceae and Fabaceae. Branch lengths reflect the estimated number of amino acid substitutions per site, with PsGALS2 showing slightly longer branches than PsGALS3, indicating greater sequence divergence. Overall, the tree shows that GALS proteins have experienced lineage-specific expansions and diversification while keeping a conserved structure within GT92.

**Fig. 4:**
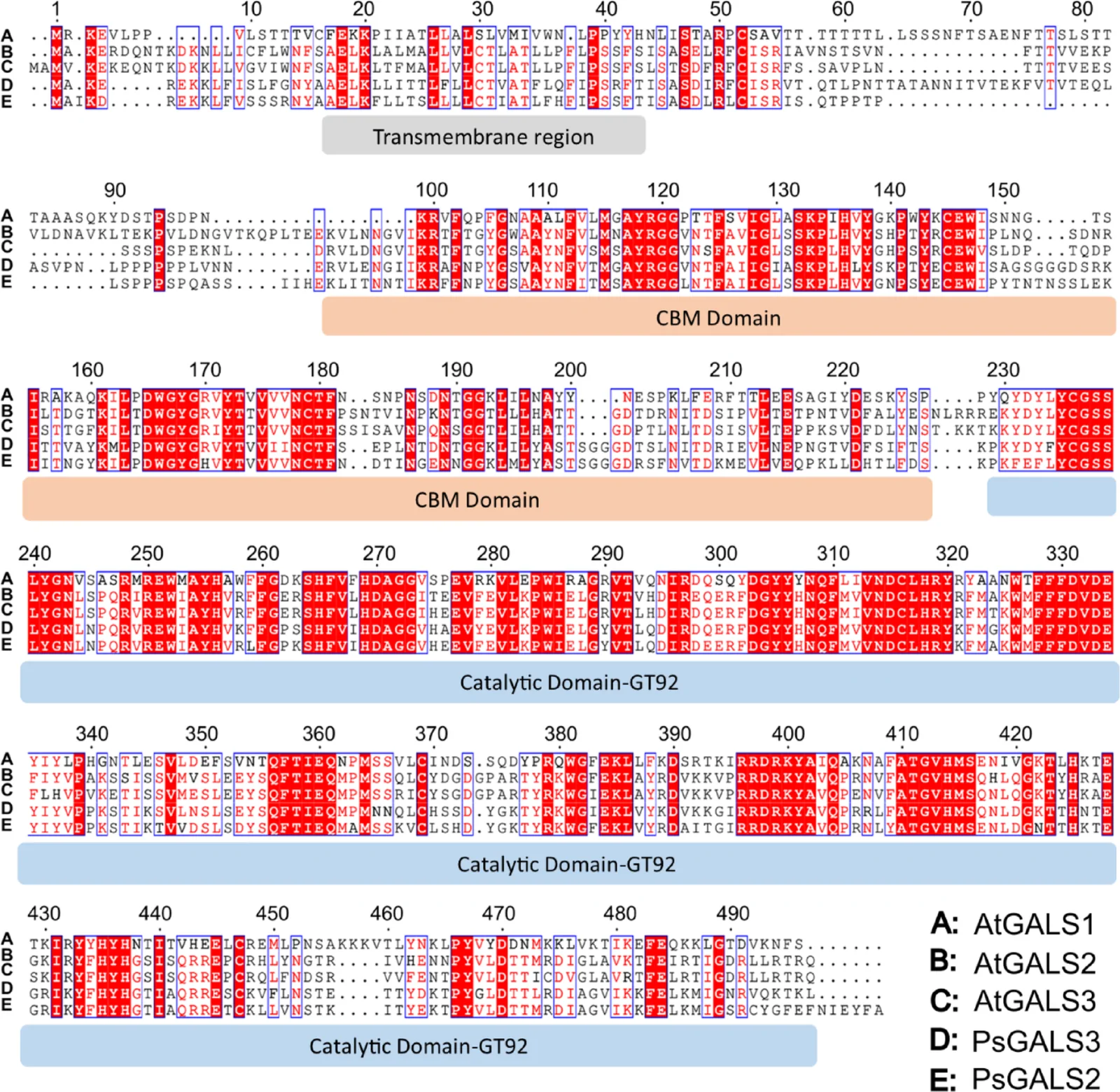
Multiple sequence alignment of the GT92 GALS proteins. Sequence similarity is shown by a white-to-red gradient background, with dark red indicating residues with a global similarity of ≥ 0.7. Identical residues are highlighted in red font. Highly conserved positions with strong local agreement are marked by blue-framed boxes. The predicted transmembrane segment is indicated by a grey bar. Domain annotations were obtained from Prabhakar et al. (2023) and CATH superfamily classifications: the N-terminal β-sandwich/Jelly-roll fold (CATH 2.60.40) is shown in brown, and the C-terminal GT-A fold catalytic domain (CATH 3.90.550) is shown in light blue

Multiple sequence alignment revealed strong conservation of key structural and catalytic motifs in GT92 galactan synthases. The domain architecture, depicted in Fig. 4, is based on Prabhakar et al. (2023) and UniProt annotations for AtGALS proteins. According to CATH classifications, GALS proteins include two primary domains: an N-terminal β-sandwich/Jelly-roll fold (CATH 2.60.40) and a C-terminal GT-A fold catalytic domain (CATH 3.90.550). Prabhakar et al. (2023) identified the N-terminal β-sandwich/Jelly-roll region as a CBM95 carbohydrate-binding module that interacts specifically with the RG-I backbone in *Populus trichocarpa*. Alignment results showed that the N-terminal transmembrane helix and the C-terminal GT-A fold catalytic domain are highly conserved across all Arabidopsis and pea sequences, consistent with their predicted Golgi localisation as type II membrane proteins. Conversely, the CBM-like domain exhibited moderate conservation with species-specific variation, suggesting functional divergence in carbohydrate recognition. Among Arabidopsis proteins, AtGALS2 and AtGALS3 were the most similar, while AtGALS1 was more divergent. The two pea paralogs also showed considerable similarity, with PsGALS2 being slightly more divergent. Overall, the alignment supports conserved catalytic activity with lineage-specific adaptations in substrate-interaction regions.

### Both *PsGALS *are expressed in aerial organs, but only *PsGALS3* is auxin-inducible

**Fig. 5:**
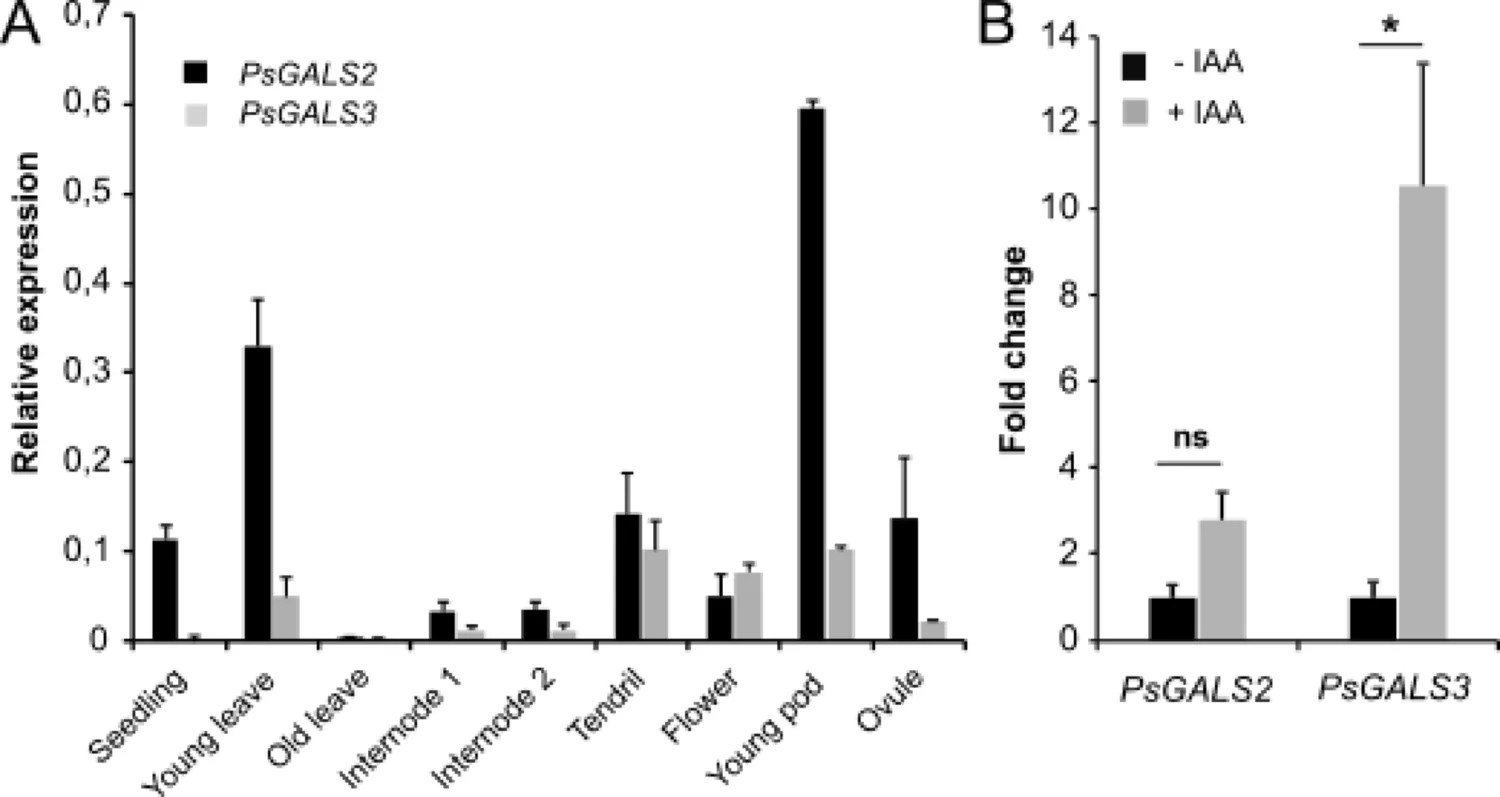
Expression analysis of *PsGALS2* and *PsGALS3.* **A** RT-qPCR analysis of eight aerial organs, including whole seedlings. Expression levels were normalised to three reference genes. **B** Auxin inducibility. Expression of *PsGALS2* and *PsGALS3* following IAA lanolin paste or application in comparison to pure lanolin paste control. Unlike *PsGALS2*, *PsGALS3* was significantly upregulated by lanolin-containing IAA. The fold change in expression compared to lanolin-only treatment. Bars represent mean efficiency-corrected normalised expression, and error bars indicate SE calculated from biological replicates (ns-not significant, * *P* < 0.05, t-test)

To characterise the expression patterns of the two pea *GALS* genes, we performed RT-qPCR on eight aerial organs and whole seedlings (Fig. 5A). *PsGALS3* was expressed across all aerial organs analysed, with the highest transcript abundance in tendrils and the lowest in mature leaves. *PsGALS2* showed generally higher expression than *PsGALS3*, with particularly high transcript levels in young pods. Overall, both genes were expressed throughout the aerial organs examined, with transcript abundance tending to be higher in younger, actively growing tissues, although no tissue-specific expression peak was observed. To assess auxin responsiveness, transcript levels were compared in second internodes treated with IAA-containing or lanolin paste alone (Fig. 5B). *PsGALS3* expression was significantly induced by IAA treatment, whereas *PsGALS2* exhibited only a modest response.

### Silencing of the *PsGALS3* gene in plants using VIGS results in severe growth defects

**Fig. 6:**
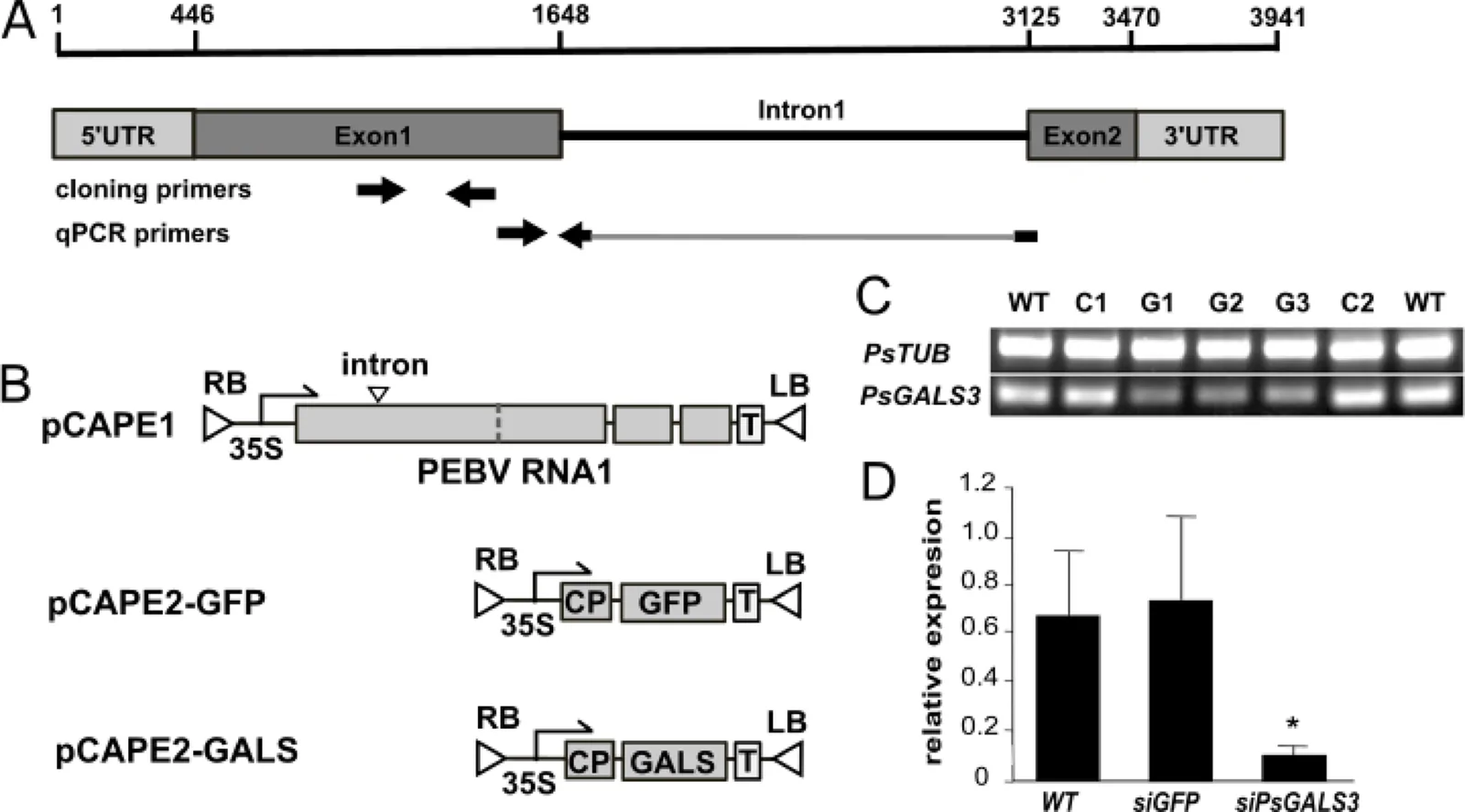
VIGS vector construction and determination of *PsGALS3* silencing efficiency **A** The scheme of the *PsGALS3* gene with depicted exons and an intron, and primers used for cloning of VIGS constructs and PCR. **B** *Pea early browning virus* (PEBV) RNA1 and RNA2 were inserted into pCAPE1 and pCAPE2, respectively. pCAPE2 carried genes encoding the viral coat protein (CP) and a *PsGALS fragment. GFP* insertion was used as a virus (vector) control (pCAPE-GFP; *siGFP*). **C** Semiquantitative PCR analysis of silencing efficiency with β-tubulin as a housekeeping control (top panel) showed a downregulation of *PsGALS3* (lower panel) in silenced lines (G1, G2, G3) compared to WT (WT1, WT2) and control *siGFP* (C1, C2) plants. **D** RT-qPCR showed a significant reduction in *PsGALS3* mRNA levels in the *siPsGALS3* plant. The level of expression was calculated relative to the TFIIA gene (mean ± SD of three biological replicates; *P < 0.05, t-test)

To elucidate the consequences of the loss of galactan synthesis on pea elongation, we used virus-induced gene silencing (VIGS) to downregulate the auxin-inducible *PsGALS3*. The pea early browning virus (PEBV) binary vector system was used to complement VIGS, with a 463 bp cDNA fragment of *PsGALS3* inserted into pCAPE 2, which contains genes encoding the virus coat protein (Constantin et al. 2004, Fig. 6A, B). GFP gene insertion served as a control for the empty virus (*siGFP*). Using these constructs, we performed the silencing experiment as described previously (Grønlund et al. 2010). The second internodes, collected three weeks after decapitation and 10 days after infiltration, were analysed for PsGALS3 expression using semi-quantitative reverse transcriptase PCR (Semi-qRT-PCR) and real-time PCR (RT-qPCR) to validate the silencing efficiency. The expression data showed that *PsGALS3* was successfully silenced to approximately 15% of its normal expression (Fig. 6C, D).

**Fig. 7:**
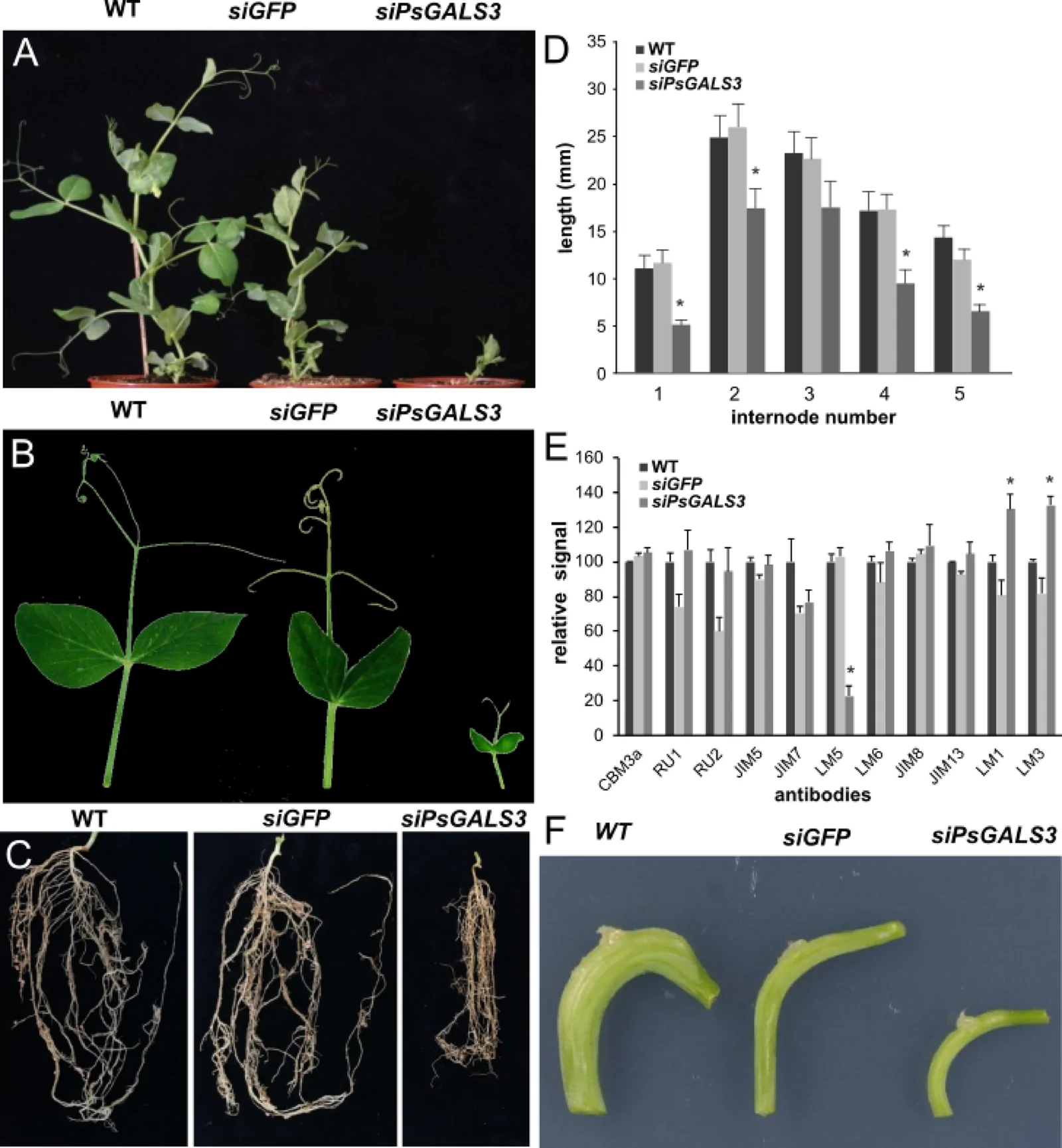
Phenotypes of *PsGALS3*-silenced plants*.* **A** Phenotypes of pea plants three weeks after virus inoculation. The *PsGALS3-*silenced plant showed a dwarf phenotype compared to the *siGFP* control and wild type. **B** *siPsGALS3* plants showed defects in aerial organ expansion and **C** reduced root system. **D** Quantification of internode length. *PsGALS3-*silenced plants significantly reduced the length of internodes (mean ± SEM of 12 biological replicates; * P < 0.05; t-test). **E** MAPP analysis of *siPsGALS3* plants. Profiling showed the relative abundance of CDTA-extractable cell wall epitopes. The LM5 signal was decreased significantly in the silenced plant, while extensin-related LM1 and LM3 got upregulated (normalised to 100% of WT signal; mean ± SD of three biological replicates; *P < 0.05; t-test). **F** Effect of galactan loss on auxin-induced bending capacity. The auxin-containing paste induced bending of the 1st internode in *siPsGALS3* plants. At least 7 independent plants were analysed

We analysed the phenotypes of si*PsGALS3* plants and compared them with those of non-treated wild-type and *siGFP* control plants. The si*PsGALS3* plants were severely stunted compared to the empty virus controls (Fig. 7), featuring up to 50% reductions of internode length (Fig. 7D). In addition to shortened internodes, *siPsGALS3* plants produced smaller leaves, shorter tendrils, and a reduced root system (Fig. 7B, C).

### *siPsGALS3* plants have significantly reduced galactan epitopes but largely remain sensitive to auxin paste

To assess silencing efficiency at the level of cell wall component abundance, we analysed cell wall composition of stems from silenced plants using MAPP and selected pectin- and glycoprotein-related mAbs. The results confirmed a strong reduction in LM5 galactan epitopes in *siPsGALS3* compared to controls, to approximately 30% (Fig. 7E). Extractable cellulose, HG, and arabinan were not significantly changed. However, significant upregulation of LM3 and LM1 epitopes was noticeable. This experiment indicated the efficacy of the VIGS approach and the functional relationship between the *PsGALS3* gene and galactan synthesis. Next, we tested the auxin response capacity of the *siPsGALS3* stems*.* As in the initial experiments, we applied auxin to pea internodes, including wild-type and VIGS control plants. Although noticeably shorter, the first internodes of *siPsGALS3* plants bent overall similarly to WT and *siGFP* control plants after auxin stimulation, which suggests that auxin-induced bending was retained despite substantially reduced basal galactan levels and visibly impaired internode size (Fig. 7F).

### Visualising the effect of *PsGALS3* silencing on galactan and extensin epitopes in situ

**Fig. 8:**
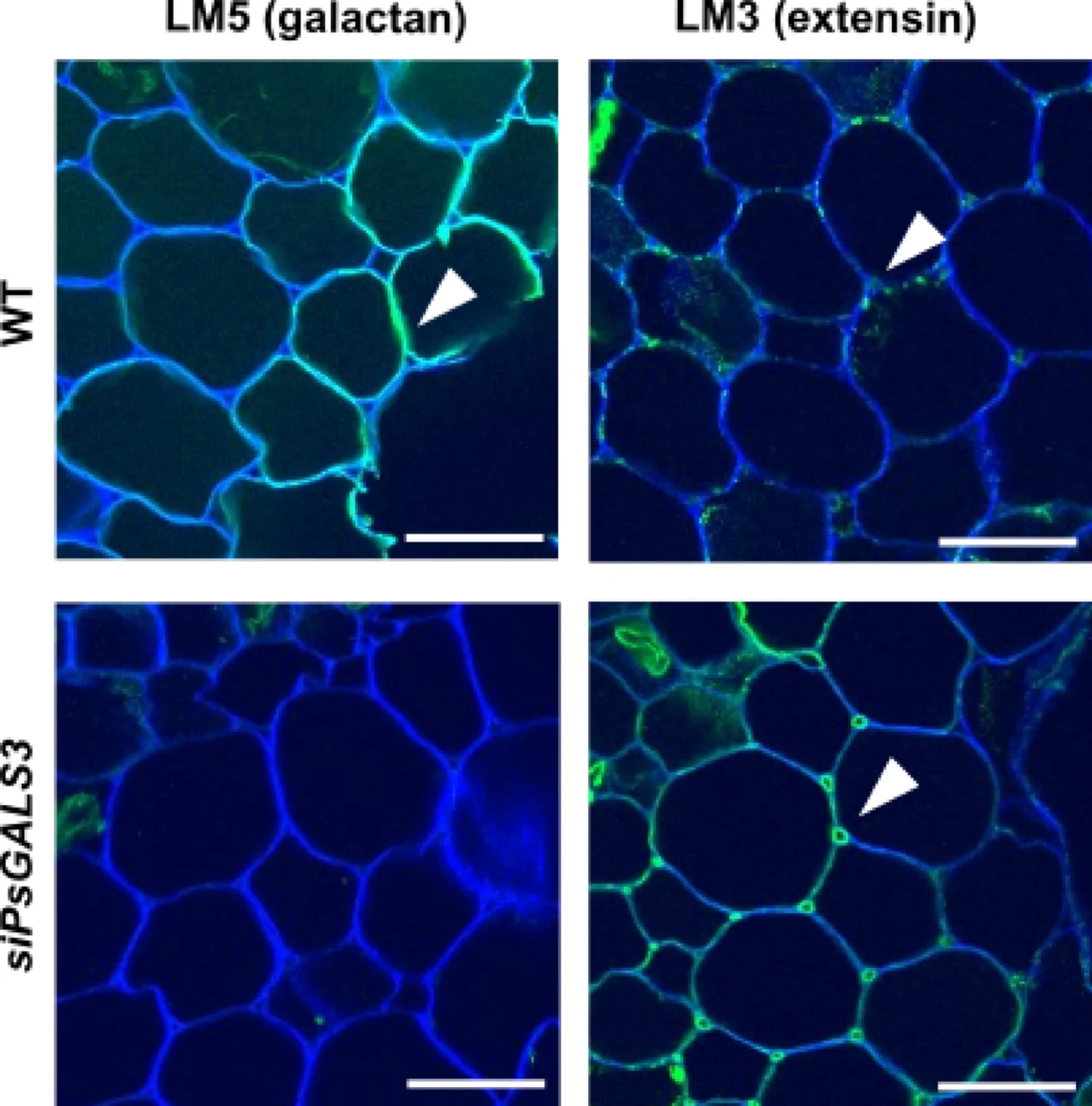
Immunolocalisation of cell wall epitopes in pea stems of wild type (WT) and *siPsGALS3* plants. Transverse sections of the second internode were stained with LM5 (galactan) and LM3 (extensin). Antibody-generated signals are shown in green, and Calcofluor White was used as a cell wall counterstain (blue). Arrowheads depict the signal. Note the loss of the LM5 signal and upregulation of the LM3 signal (triangular junctions) in the *siPsGALS3* plant. Scale bar = 20 μm

Comparing MAPP data on auxin-induced stem curvature and si*PsGALS3* pea plants revealed a downregulation and upregulation of galactan and extensin epitopes, respectively. To confirm this inverse correlation in situ, we performed immunolocalisation on the vibratome-generated transversal pea stem sections. In WT plants, probing with LM5 mAb showed only labelling in pith parenchyma cells. The LM3 signal was generally weak and confined to small patches. In silenced plants, LM5 labelling was visibly reduced; on the other hand, LM3 labelling increased, and the signal was mainly restricted to triangular cell junctions in the parenchyma tissue (Fig. 8).

## Discussion

The dynamic changes in cell wall properties required to match developmental programmes involve modulating composition and the intricate spatiotemporal arrangement of various cell wall components. Cellular expansion and cell division are the primary processes that shape the plant body. It has long been proposed that a key factor determining cell wall extensibility is the reversible noncovalent interaction between hemicelluloses, specifically xyloglucan, and cellulose microfibrils (Cosgrove 2024). This interaction is linked to auxin-triggered expansin activation via apoplast acidification (Fendrych et al. 2016). Other cell wall components are also crucial in making cell walls extensible while providing strength and supporting load-bearing functions. Pectin side chains and HG modifications, especially demethylation, play a vital role in this process.

Galactan epitopes are specifically localised to the Arabidopsis root tip’s elongation zone by immunohistochemistry and glycan profiling (McCartney et al. 2003; Wilson et al. 2015) as well as in a monocot species such as rye (Petrova et al. 2022). In line with this, in our setup replicating pea tropism, galactan was upregulated on the more elongated side of the auxin-induced curvature, which is in line with the auxin inducibility of *PsGALS3*.

Current knowledge of pectic galactan synthesis and function is largely derived from *Arabidopsis thaliana*, where members of the GT92 family (AtGALS1–3) function as β-(1→4)-galactan synthases required for RG-I galactan biosynthesis (Liwanag et al. 2012; Ebert, 2018; Laursen et al. 2018; Prabhakar et al. 2023). Although galactan has been associated with cell elongation based on its spatial distribution in expanding tissues, *Arabidopsis gals* mutants exhibit only relatively mild developmental phenotypes, except for altered responses to salt stress (Yan et al. 2021) or in cold acclimation (Takahashi et al. 2024).

Early research in pea revealed that galactan is synthesised as part of a pectin-xyloglucan complex in the Golgi apparatus, which is then deposited into the primary cell wall (Abdel-Massih et al. 2003; Cumming et al. 2005). Later studies in Arabidopsis indicated that remodelling of galactan affects xyloglucan structure during cell elongation (Moneo-Sánchez et al. 2019), and auxin-driven tissue expansion depends on the dynamic remodelling of xyloglucan (Velasquez et al. 2021). In this context, the auxin-induced increase in galactan accumulation observed in this study, together with previous evidence for a pectin–xyloglucan complex in pea (Abdel-Massih et al. 2003; Cumming et al. 2005) and the role of xyloglucan remodelling during auxin-dependent growth (Moneo-Sánchez et al. 2019; Velasquez et al. 2021), is consistent with coordinated remodelling of pectic galactan and xyloglucan during stem elongation. Such remodelling may facilitate the reorganisation of primary cell wall networks required for rapid auxin-induced cell expansion. Alternatively, galactan may modify cell wall mechanics by influencing interactions between pectin and other wall polymers, thereby affecting the gelation properties of pectin (Willats et al. 2006; Gao et al. 2023). However, the precise role of galactan in contributing to primary cell wall extensibility remains to be elucidated.

Unlike in Arabidopsis, downregulating galactan synthesis has severe effects on pea growth, including inhibited organ expansion. VIGS is generally used in plants with limited genetic resources; however, the transient nature of gene downregulation, residual gene expression, potential co-silencing of other genes, and variability in silencing efficiency remain confounding factors. These factors or compensatory mechanisms could account for the observation that auxin remained able to bend the internodes of *siPsGALS3* plants. Another possibility is that galactan accumulation accompanies, rather than determines, auxin-mediated elongation.

It is important to bear in mind that not all type-I cell walls are the same. We have previously presented evidence that the cell wall of members of the fabid clade displays separability differences compared with members of the malvid clade to which Arabidopsis belongs (Fangel et al. 2024). In other words, if differences in polysaccharide separability are regarded as evidence for cell wall architectural differences, then Arabidopsis is not a valid model for pea in all aspects. Notably, galactan was one of the polymers that showed differences in partitioning (Fangel et al. 2024).

Extensins are *O*-glycosylated proteins that belong to the hydroxyproline-rich glycoproteins (HRGPs) superfamily. Extensin sidechains on hydroxyproline consist of one to four arabinose residues, while serine may carry a single galactose residue (Velasquez et al. 2011; Saito et al. 2014; Moussu et al., 2023; Moussu and Ingram, 2023). Although our data suggest that extensins are upregulated upon loss of galactan, the functional relationship between these two cell wall components remains unclear, and we cannot exclude that the increased labelling observed in immunolocalisation is, in part, due to better exposure of the extensin in the wall with truncated galactans (Marcus et al. 2008). The non-ionic interaction between extensin and pectin has been identified (Nuñez et al. 2009), and its importance for cell wall integrity has been demonstrated (Velasquez et al. 2011). Numerous previous studies suggest that extensins may play a general role in the cell wall response to stress (Houston et al. 2016). For example, extensin-coding genes are upregulated during mechanical stress in tension wood formation or in response to pathogen attacks (Wei and Shirsat 2006). The mechanism by which cross-linking of extensins through tyrosine residues strengthens the cell walls has also been proposed (Cannon et al. 2008). This is further supported by our observation of extensins in the cell corners of parenchyma tissue in *siPsGALS3* plants. Numerous cell wall-associated kinases and integrity-sensing systems have been shown to sense pectin-mediated cell wall homeostasis (Gigli-Bisceglia et al. 2020; Moussu et al., 2023; Rößling et al. 2024). However, no galactan-specific receptor-like kinase has yet been identified, so the functional connection between the content of galactan and extensin structures and any putative compensatory mechanism remains obscure.

Together, our findings suggest that the contribution of galactan to primary cell wall function is species dependent and may reflect differences in cell wall architecture between Fabaceae and Brassicaceae. More extensive molecular and genetic studies on peas should now be facilitated by the availability of the pea draft genome (Liu et al. 2024). In addition, more research on the interactions among various cell wall components *in muro* is needed to accurately understand the underlying mechanism of cell elongation.

## Material and methods

### Plant material and growth conditions

*Pisum sativum* cv. Kelvedon Wonder seeds (Frøbutikken, Denmark) were sterilised with absolute hypochlorite for 3 min by shaking, rinsed in sterile water at least five times, and left in the last rinse for 1 h. They were then placed on wet tissue inside a sterilised plastic box in the dark at room temperature for five days to germinate. Seedlings that germinated synchronously with roots around 3–4 cm were transferred to soil (Pindstrup Mosebrug A/S, Denmark), with one seedling per 11 cm pot. Plants were grown at 22 °C/20 °C under a 16-h/8-h day/night cycle in a growth chamber illuminated with fluorescent light (200 μmol m-2 s-1).

### Auxin-induced stem curvature

The setup has been described by Velasquez et al. (2021). Three-week-old pea plants were decapitated at the second internode counting from the tip. Lanolin paste containing indole−3-acetic acid (IAA, Sigma-Aldrich) at 10 mg/g was applied to one side of the stem of the second internode. The wound surface introduced by decapitation was covered by lanolin without IAA for protection from dehydration. Curvature or straight stems were harvested after 3 days of treatment. The curvature was dissected from the middle to separate the longer and shorter sides, and a pool of three curvatures was used for further analysis of cell wall composition. Additionally, after 24 h with or without treatment, RNA was isolated to quantify *PsGALS2* and *PsGALS3* in auxin-induced stem curvature.

### Preparation of alcohol insoluble residues

Plant materials were first homogenised using TissueLyser (Sigma-Aldrich) with a metal bead at maximum speed. Alcohol-insoluble residue (AIR) was prepared following sequential extraction with 70% ethanol, methanol: chloroform at 1:1 (v/v), and absolute acetone. Each solvent was added to the samples at 6:1 (v/v) and mixed thoroughly by vortexing. Each extraction lasted 10 min with shaking, then the samples were centrifuged at 14,000 rpm for 10 min. The supernatant was discarded, and the samples were left to dry in the fume hood. AIR from pea stem curvature was used in monosaccharide analysis and epitope detection chromatography. The AIR from a virus-silenced pea plant stem was used in comprehensive microarray polymer profiling (MAPP).

### Microarray polymer profiling

MAPP analysis was conducted as previously described (Ebert et al. 2018). AIR samples were extracted sequentially with two solvents: 50 mM CDTA and 4 M NaOH with 1% (v/v) NaBH4 at 30:1 (μl/mg). After 2 h of extraction with shaking, followed by centrifugation at 4000 rpm, supernatants were spotted onto nitrocellulose membranes in four replicates and four dilutions at ratios of 1:2, 1:6, 1:18, and 1:54 [v/v]. The microarray was probed with cell wall-specific probes, and the binding intensity was quantified as MAPP data. The microarray was probed with cell wall-specific probes, and the binding intensity was quantified as MAPP data. Data were presented as line charts generated by plotting individually scaled MAPP values for each probe. For each chart, the maximum signal intensity among the analysed probes was set to 100, and the remaining signals were scaled proportionally relative to this value.

### Monosaccharide composition analysis

Monosaccharide analysis was carried out as previously described (Ebert et al. 2018). 1 mg of the AIR sample was hydrolysed in 2 M trifluoroacetic acid (TFA) for 1 h at 120 °C and dried using vacuum spin to remove TFA. Residuals resolved in H_2_O and applied to ICS 3000 (Dionex) with a PA20 column performing high-performance anion-exchange chromatography with pulsed amperometric detection (HPAEC-PAD). Monosaccharide standards included L-Fuc, L–Rha, L-Ara, D-Gal, D-Glc, D-Xyl, D-GalUA, and D-GlcA (Sigma-Aldrich). Three biological replicates were used for the analysis.

### Epitope detection chromatography

Epitope detection chromatography (EDC) was carried out as previously described (Cornuault et al. 2014). AIR samples were extracted using CDTA. 50 μl of the extraction was diluted in 2.5 ml H2O and eluted through a 1 ml Hi-Trap ANX FF anion-exchange column (GE Healthcare) with Na_2_CO_3_ buffer. 100 μl of fraction aliquots were incubated overnight at 4 °C in a microtitre plate and probed with cell wall-specific probes. The absorbance intensity was determined at 450 nm using an ELISA plate reader (BioTek).

### Embedding and sectioning

Vibratome sectioning (Fig. 8): Transverse sections were prepared from approximately 1-cm stem segments embedded in 8% agarose and sectioned with a Leica VT1000s vibratome. The internode segments were preserved in 70% ethanol and then rehydrated in PBS before being embedded in agarose. The blocks were sectioned to generate 200 μm-thin slices. The sections were collected in a 24-well microplate. For resin cross-section preparation (Fig. 5), 1 cm internode segments from IAA-induced curved stems and untreated control stems were fixed in 4% (w/v) paraformaldehyde in PBS for 2 h under vacuum infiltration. Following fixation, the samples were washed three times in PBS for 10 min each and dehydrated through a graded ethanol series (25, 50, 70, 90, 96 and 100%). Samples were then infiltrated with LR White medium-grade acrylic resin by sequential incubation in ethanol:resin mixtures (2:1, 1:1, and 1:2, v/v) for 2 h each, followed by two changes of 100% LR White resin, each for 2 h. The samples were left overnight at 4 °C in fresh 100% LR White resin. The infiltrated samples were transferred into gelatin capsules filled with fresh LR White resin and polymerised at 60 °C for 24 h. The blocks were trimmed and sectioned with a glass knife on an Ultratome Supernova (Reichert-Jung, Vienna, Austria) to obtain 1 µm-thick sections.

### Immunolocalisation

Sections (vibratome or resin adhered to glass slides) were blocked with 5% skimmed milk powder in PBS for 30 min, incubated with primary antibodies at a 1:100 dilution for 1 h, and washed three times for 10 min each. The secondary antibody, goat anti-rat IgG AlexaFluor 488 and 555 (Invitrogen), was prepared at a 1:500 dilution and incubated for 1 h. After three PBS washes, sections were counterstained with Calcofluor White (Sigma-Aldrich) at 0.1 mg/ml for 10 min. Immunolabeled sections were mounted in CitiFluor (Agar Scientific), and sections were imaged using a Leica DM5500 B epifluorescence microscope (Leica Microsystems, Wetzlar, Germany).

### Laser Scanning Confocal Microscopy

Imaging was performed using a Leica SP5 confocal microscope with UV diode (405 nm) and Argon (488 nm) lasers. Imaging of the longitudinal section was performed using an Olympus BX61 microscope equipped with epifluorescence irradiation. Images were processed in GIMP2 only to enhance contrast and brightness. All scans were treated equally.

### DNA cloning

Total RNA was extracted from 100 mg of leaf tissue using the Spectrum™ Plant Total RNA Kit (Sigma-Aldrich) and used as a template for first-strand cDNA synthesis with SuperScript™ SuperMix (Thermo Fisher Scientific). The resulting cDNA served as the template for amplification of a 463 bp fragment of *PsGALS3* using primers PsGALS3-C-F and PsGALS3-C-R. The VIGS vector pCAPE2-USER was generated by replacing the GFP coding sequence in pCAPE2-GFP (Constantin et al. 2004) with the USER cloning cassette sequence GCTGAG GCAATTTAAATACCCTCAGC. Prior to cloning, pCAPE2-USER was linearised with *SwaI* (New England Biolabs), and USER-compatible overhangs were generated using Nb.BbvCI (New England Biolabs). The amplified *PsGALS3* fragment was then inserted into pCAPE2-USER by USER cloning (Nour-Eldin et al. 2010) to generate the VIGS construct pCAPE2-PsGALS3. Primer sequences are listed in Supplementary Table 1.

### Virus-induced gene silencing

Virus-induced gene silencing was performed as described earlier (Grønlund et al. 2010). The pea early browning virus (PEVB) system uses binary vectors derived from pCAMBIA 1300, named pCAPE1 and pCAPE2. These vectors were separately transformed into the Agrobacterium strain GV3101 and cultured. The bacteria were collected by centrifuging at 3500 g once the culture reached an optical density of 1.2–1.5 at OD550, then resuspended in infiltration buffer (10 mM NaCl, 1.75 mM CaCl2, 100 μM acetosyringone). The cultures containing pCAPE1 and pCAPE2 were mixed 1:1. This mixture was infiltrated into the abaxial side of leaves of 2-week-old pea plants using a 1 ml syringe. pCAPE2 containing GFP was used as a control. Ten days post-infiltration, pea plants were decapitated at the nodules formed after infiltration. New shoots emerged from the bottom of the plants. Samples for analysis were collected three weeks after decapitation. Photographs of VIGS-silenced pea plants were taken with a Canon EOS 20D camera equipped with an EFS 60 mm lens. Internode length were measured using ImageJ software (https://imagej.nih.gov/ij/). Internodes were numbered from the most apical (1) to the basal (5).

### Gene expression analysis

For semi-quantitative PCR (Semi-qRT-PCR), RNA was extracted from 100 mg of leaf tissue (SpectrumTM Plant total RNA kit, Sigma Aldrich). 500 ng RNA was used as a template for cDNA synthesis (Superscript supermix). For semi-qRT-PCR, a 188 bp fragment of PsGALS3 was amplified with primers PsGALS3-sqF and PsGALS3-sqR, and a 573 bp fragment of β-tubulin cDNA (accession no. NM_001427110.1) with primers TUB-F1 and TUB-R1. PsGALS3 Semi-qRT-PCR primers were predicted to span one intron based on the corresponding *Arabidopsis* gDNA sequences At5g44670 and At4g20170, while tubulin Semi-qRT-PCR primers span two introns in pea gDNA (X54844). PCR conditions were optimised, and were run for 30 cycles of 30 s at 94 °C, 30 s at 61 °C, and 40 s at 72 °C. PCR products were loaded into the same gel by first loading 14 μl of 188 bp *PsGALS3* fragment, electrophoresing for 5 min, followed by loading 14 μl of 573 bp tubulin fragment. For RT-qPCR on silenced plants, RNA was extracted from 100 mg of leaf tissue (SpectrumTM Plant total RNA kit). 3.5 μg RNA was treated with DNA-free Turbo (Ambion), and 850 DNAse-treated RNA was used as template for cDNA synthesis (iscriptTM cDNA synthesis kit, Bio-Rad). Transcription factor 2a (TFIIA; accession no. XM_051028068.1) was selected as a housekeeping reference and amplified with primers TFIIA-F1 and TFIIA-R1. Analysis of expression in organs: Total RNA was extracted from three-day-old seedlings and various organs, namely young leaves, old leaves, internodes I and II, flowers (without sepals), young pods (without ovules), ovules, and tendrils using the Quick-RNA™ Plant Miniprep Kit (Zymo Research, USA) with DNase treatment. First-strand cDNA was synthesised from 1 µg of total RNA from each organ using the RevertAid First Strand cDNA Synthesis Kit (Thermo Fisher Scientific, USA) with a mixture of oligo(dT) and random primers.

Gene expression and auxin inducibility: Quantitative real-time PCR (qRT–PCR) was performed using the FastStart Essential DNA Green Master Mix (Roche) on a LightCycler® 96 system. The reference genes phosphoprotein phosphatase 2A (PP2A; accession no. NM_001427333.1), TFIIA, and β-tubulin were selected based on their reported expression stability (Die et al. 2010; Knopkiewicz and Wojtaszek 2019). Amplification specificity was confirmed by melt-curve analysis. Ct values from three technical replicates were averaged for each of two biological replicates. For expression analysis of aerial pea organs, three biological replicates were used, whereas four biological replicates were analysed for IAA-treated stem samples. Relative expression levels of *PsGALS3* and *PsGALS2* were calculated using an efficiency-corrected method (RQ=E^−Ct^, where RQ represents relative quantities and E is the PCR amplification efficiency), and normalised to the geometric mean of the reference genes. Standard error (SE) was calculated from efficiency-corrected normalised expression values obtained from three biological replicates. See Supplementary Table 2 for details of the primers used and Supplementary Fig. 1 for the primer positions within the *PsGALS2* gene.

## Supplementary Information

Below is the link to the electronic supplementary material.

Supplementary Material 1

## Data Availability

No datasets were generated or analysed during the current study.
