# Supplementary material for: Loss of galactan synthesis in pea (Pisum sativum) causes defects in organ expansion and is associated with increased extensin content
Source: Plant Mol Biol. 2026 Sep 9;116(5):94. doi: 10.1007/s11103-026-01759-x (PMC13558309; doi:10.1007/s11103-026-01759-x)
Supplement: Supplementary file 1 [file 11103_2026_1759_MOESM1_ESM.docx]

**Supplementary Table 1**: List of primers

| **Targets** | **Primer names** | **Sequences (5′→3′)** | **Applications** |
| --- | --- | --- | --- |
| PsGALS2 | PsGALS2-q-F | ATGCTTCAACATCAGGTGGC | RT-qPCR |
|  | PsGALS2-q-R | TCCCATACAAAGATGATCCACAGT |  |
| PsGALS3 | PsGALS3-C-F | GGCAATTUCTGTACCTTCTCGGAGCCGC | USER cloning |
|  | PsGALS3-C-R | GGGTATTUCAAAGAAGAACATCCATTTCCCC |  |
|  | PsGALS3-Sq-F | CGGTGCTCAATTCGCTATCGG | Semi-qRT-PCR |
|  | PsGALS3-Sq-R | TAGCCTACGTGGTTGCACTGC |  |
|  | PsGALS3-q-F | ATCGGTGCTCAATTCCCTATC | RT-qPCR |
|  | PsGALS3-q-R | AGCTTTTCAATTCCCCATTTCCTAT |  |
| β-tubulin | TUB-F1 | ACTGTGATTGCTTGCAGGGT | Reference genes for RT-PCR |
|  | TUB-R1 | ACATAGCAGATGCGGTGAGG |  |
|  | TUB-F2 | TGCAGATGAGGTTATGGTTCTTGA |  |
|  | TUB-R2 | TGTTACACCAGACATAGTTGCTG |  |
| TFIIA | TFIIA-F1 | GCAACCTCCTTCTCCTTGGAT |  |
|  | TFIIA-R1 | TCTTCCCGTCCTTCCACATAA |  |
|  | TFIIA-F2 | CTCAGCAAGATGGAGCTGGT |  |
|  | TFIIA-R2 | CCAAGTCTGCGGGCATTTTC |  |
| PP2A | PP2A-F1 | AAGTGACCAAGTTCTCCCGC |  |
|  | PP2A-R1 | GACCGGTGCCATTCCCATTA |  |

Note: C: cloning primers; Q: RT-qPCR primers; S: semi-quantitative RT-PCR; F: forward and R: reverse primers.

**Supplementary Figure 1:** The structure of the *PsGALS2* gene with the indicated position of the primers used for RT-qPCR

**Supplementary Table 2:** List of used antibodies/probes

| **Probe** | **Cell wall component** | **Recognized epitope** | **Reference** | **Used to profile IAA-stimulated internodes** |
| --- | --- | --- | --- | --- |
| **CBM3a** | Cellulose/xyloglucan | Crystalline cellulose (cellulose I)/xyloglucan; type-A carbohydrate-binding module | McCartney et al. (2004) |  |
| **RU1** | Rhamnogalacturonan-I (RG-I) | Long stretches of unbranched RG-I backbone (alternating Rha–GalA repeats) | Ralet et al. (2010) |  |
| **RU2** | Rhamnogalacturonan-I (RG-I) | Shorter stretches of unbranched RG-I backbone | Ralet et al. (2010) |  |
| **JIM5** | Homogalacturonan (HG) | Low methyl-esterified / partially de-esterified HG | Knox et al. (1990); Willats et al. (2000); Clausen et al. (2003) | * |
| **JIM7** | Homogalacturonan (HG) | Partially methyl-esterified HG | Knox et al. (1990); Willats et al. (2000); Clausen et al. (2003) | * |
| **JIM8** | Arabinogalactan proteins (AGPs) | AGP carbohydrate epitope (exact structure not fully defined) | Pennell et al. (1991) | * |
| **JIM13** | Arabinogalactan proteins (AGPs) | AGP glycan epitope containing glucuronic acid-associated structures | Knox et al. (1991); Møller et al. (2008) | * |
| **LM1** | Extensins (HRGPs) | Extensin glycoprotein epitope (precise structure unknown) | Smallwood et al. (1994) | * |
| **LM3** | Extensins (HRGPs) | Extensin-related/not defined | Feng et al. (2014) | * |
| **LM5** | RG-I | (1→4)-β-D-Galactan side chains | Jones et al. (1997) | * |
| **LM6** | RG-I | (1→5)-α-L-Arabinan/AGP-related | Willats et al. (1998) | * |
| **LM10** | Xylan | Unsubstituted or sparsely substituted β-(1→4)-xylan | McCartney et al. (2005) | * |
| **LM13** | RG-I | Linear (1→5)-α-L-arabinan | Møller et al. (2008) | * |
| **LM14** | Arabinogalactan proteins (AGPs) | Glucuronic acid-containing AGP epitope | Møller et al. (2008) | * |
| **LM15** | Xyloglucan | XXXG motif of xyloglucan | Marcus et al. (2008) | * |
| **LM21** | Heteromannans | Mannan/glucomannan/galactomannan backbone | Marcus et al. (2010) | * |

**References for Supplementary Table 2**

Clausen MH, Willats WGT, Knox JP (2003) Synthetic methyl hexagalacturonate hapten inhibitors of anti-homogalacturonan monoclonal antibodies LM7, JIM5 and JIM7. Carbohydr Res 338:1797–1800

Feng T, Nyffenegger C, Højrup P, Vidal-Melgosa S, Yan KP, Fangel JU, Meyer AS, Kirpekar F, Willats WGT, Mikkelsen JD (2014) Characterization of an extensin-modifying metalloprotease: N-terminal processing and substrate cleavage pattern of *Pectobacterium carotovorum* Prt1. Appl Microbiol Biotechnol 98:10077–10089

Jones L, Seymour GB, Knox JP (1997) Localization of pectic galactan in tomato cell walls using a monoclonal antibody specific to (1→4)-β-D-galactan. Plant Physiol 113:1405–1412

Knox JP, Linstead PJ, King J, Cooper C, Roberts K (1990) Pectin esterification is spatially regulated both within cell walls and between developing tissues of root apices. Planta 181:512–521

Marcus SE, Verhertbruggen Y, Hervé C, Ordaz-Ortiz JJ, Farkas V, Pedersen HL, Willats WGT, Knox JP (2008) Pectic homogalacturonan masks abundant sets of xyloglucan epitopes in plant cell walls. BMC Plant Biol 8:60

Marcus SE, Blake AW, Benians TAS, Lee KJD, Poyser C, Donaldson L, Leroux O, Rogowski A, Petersen HL, Boraston AB, Gilbert HJ, Willats WGT, Knox JP (2010) Restricted access of proteins to mannan polysaccharides in intact plant cell walls. Plant J 64:191–203

McCartney L, Gilbert HJ, Bolam DN, Boraston AB, Knox JP (2004) Glycoside hydrolase carbohydrate-binding modules as molecular probes for the analysis of plant cell wall polymers. Anal Biochem 326:49–54

McCartney L, Marcus SE, Knox JP (2005) Monoclonal antibodies to plant cell wall xylans and arabinoxylans. J Histochem Cytochem 53:543–546

Møller I, Marcus SE, Haeger A, Verhertbruggen Y, Verhoef R, Schols H, Ulvskov P, Mikkelsen JD, Knox JP, Willats WGT (2008) High-throughput screening of monoclonal antibodies against plant cell wall glycans by hierarchical clustering of their carbohydrate microarray binding profiles. Glycoconj J 25:37–48

Pennell RI, Janniche L, Kjellbom P, Scofield GN, Peart JM, Roberts K (1991) Developmental regulation of a plasma membrane arabinogalactan protein epitope in oilseed rape flowers. Plant Cell 3:1317–1326

Ralet M-C, Tranquet O, Poulain D, Moïse A, Guillon F (2010) Monoclonal antibodies to rhamnogalacturonan I backbone. Planta 231:1373–1383

Smallwood M, Yates EA, Willats WGT, Martin H, Knox JP (1996) Immunochemical comparison of membrane-associated and secreted arabinogalactan-proteins in rice and carrot. Planta 198:452–459

Willats WGT, Limberg G, Buchholt HC, van Alebeek G-JWM, Benen J, Christensen TMIE, Visser J, Voragen A, Mikkelsen JD, Knox JP (2000) Analysis of pectic epitopes recognised by hybridoma and phage-display monoclonal antibodies using defined oligosaccharides, polysaccharides, and enzymatic degradation. Carbohydr Res 327:309–320

Willats WGT, Marcus SE, Knox JP (1998) Generation of a monoclonal antibody specific to (1→5)-α-L-arabinan. Carbohydr Res 308:149–152
